# A transient transformation system for gene characterization in upland cotton (*Gossypium hirsutum*)

**DOI:** 10.1186/s13007-018-0319-2

**Published:** 2018-06-22

**Authors:** Haipeng Li, Kun Li, Yutao Guo, Jinggong Guo, Kaiting Miao, Jose R. Botella, Chun-Peng Song, Yuchen Miao

**Affiliations:** 1 Institute of Plant Stress Biology, State Key Laboratory of Cotton Biology, Department of Biology, Henan University, 85 Minglun Street, Kaifeng, 475001 China; 2 School of Life Science, Southwest University, No. 1, Tiansheng Road, Beibei, Chongqing, 400715 China; 3 School of Agriculture and Food Sciences, University of Queensland, Brisbane, QLD Australia

**Keywords:** *Gossypium hirsutum*, Transient transformation, *Agrobacterium*, Cotton, Gene characterization

## Abstract

**Background:**

Genetically modified cotton accounts for 64% of the world’s cotton growing area (22.3 million hectares). The genome sequencing of the diploid cotton progenitors *Gossypium raimondii* and *Gossypium arboreum* as well as the cultivated *Gossypium hirsutum* has provided a wealth of genetic information that could be exploited for crop improvement. Unfortunately, gene functional characterization in cotton is lagging behind other economically important crops due to the low efficiency, lengthiness and technical complexity of the available stable transformation methods. We present here a simple, fast and efficient method for the transient transformation of *G*. *hirsutum* that can be used for gene characterization studies.

**Results:**

We developed a transient transformation system for gene characterization in upland cotton. Using β-glucuronidase as a reporter for *Agrobacterium*-mediated transformation assays, we evaluated multiple transformation parameters such as *Agrobacterium* strain, bacterial density, length of co-cultivation, chemicals and surfactants, which can affect transformation efficiency. After the initial characterization, the *Agrobacterium* EHA105 strain was selected and a number of binary constructs used to perform gene characterization studies. 7-days-old cotton seedlings were co-cultivated with *Agrobacterium* and transient gene expression was observed 5 days after infection of the plants. Transcript levels of two different transgenes under the control of the cauliflower mosaic virus (CaMV) 35S promoter were quantified by real-time reverse transcription PCR (qRT-PCR) showing a 3–10 times increase over the levels observed in non-infected controls. The expression patterns driven by the promoters of two *G*. *hirsutum* genes as well as the subcellular localization of their corresponding proteins were studied using the new transient expression system and our observations were consistent with previously published results using *Arabidopsis* as a heterologous system.

**Conclusions:**

The *Agrobacterium*-mediated transient transformation method is a fast and easy transient expression system enabling high transient expression and transformation efficiency in upland cotton seedlings. Our method can be used for gene functional studies such as promoter characterization and protein subcellular localization in cotton, obviating the need to perform such studies in a heterologous system such as *Arabidopsis*.

**Electronic supplementary material:**

The online version of this article (10.1186/s13007-018-0319-2) contains supplementary material, which is available to authorized users.

## Background

Cotton (*Gossypium* spp.) is a very important economic crop, providing the basic resource for thousands of consumer and industrial products worldwide and its contribution to the fiber and food industries continues to grow in importance. However, cotton growers face a number of important challenges such as insect pests, weeds, and viruses that result in substantial economic losses [1, 2]. Therefore, it is important to improve the agronomic performance of cotton, and especially to enhance the insect and disease resistance of cotton plants as well as cotton fiber quality and yield.

The development of transgenic techniques for cotton in the last decade has contributed to improvements in insect resistance, fiber quality and yield [3, 4]. Despite the fact that transgenic cotton lines were first produced 30 years ago [5, 6], cotton transformation is still very difficult, inefficient and time consuming compared to other crops (e.g., rice, wheat and tomato) [3, 7]. *Agrobacterium*-mediated transformation is the preferred method to introduce transgenes into cotton and new transformation vectors, culture methodologies and techniques have had a noticeable but limited impact in the original transformation efficiency [8, 9]. Stable transformation is crucial for the implementation of the recently developed CRISPR (clustered regularly interspaced short palindromic repeats)/Cas9 genome editing technology and has been used in many animal and plant species, including cotton [10–14]. Although transient expression systems have inherent limitations, they can have important applications for gene functional studies as well as the rapid and scalable production of recombinant proteins in plants.

Most of the world’s cotton fiber is derived from *G. hirsutum*, the most widely cultivated cotton species [15]. This species is an allotetraploid with a complex genome, and most of its genes are present as multiple copies located in the At and Dt subgenomes. In recent years, two draft genome maps of *G. hirsutum* have been produced with reference genome information from two diploid progenitors, *Gossypium arboreum* and *Gossypium raimondii* [15, 16]. The new genomic information has provided a treasure trove of genes with potential applications in cotton crop improvement, however the difficult, lengthy and inefficient transformation method has left most of those genes unexplored. Transient techniques such as virus-induced gene silencing (VIGS) can be powerful tools for functional genomic studies, and it has been used in cotton [17–20]. In addition to silencing approaches, fast transient expression methods can prove valuable for functional studies in cotton but have not been reported so far.

In this study, we describe the development of a transient expression system for cotton, and used it to analyze the localization and expression patterns of two previously characterized members of the glutathione peroxidase (GPX) gene family, *GhGPX1* and *GhGPX8* [21]. The *Agrobacterium*-mediated transient transformation method is a fast and easy transient expression system for systematic gene function analysis in upland cotton.

## Methods

### Plant material

Seeds of *G. hirsutum* (TM-1 variety) were wrapped in moist absorbent cotton, placed in Petri dishes and kept in an incubator under a 14/10 h light/dark photoperiod with light intensity of 150–200 μmol m^−2^ s^−1^ at 25 °C for 3 days to germinate. The seedlings were grown in sterile culture vessels with Hoagland’s nutrient solution [22] under long-day conditions (16/8 h light/dark photoperiod) with 26/20 °C day/night temperatures. After 4 days, the seedlings (before the first true leaf appeared) were used for *Agrobacterium*-mediated transformation.

### Plasmid construction

To study the subcellular localization of *GhGPX1* and *GhGPX8*, their coding sequences (CDS) were ligated in frame with the green fluorescent protein (GFP) coding region in the *p35S*-*GFP* vector, to construct the recombinant plasmid *p35S*-*GhGPX1/8*-*GFP*. Approximately 2 kb of the promoter regions of *GhGPX1* and *GhGPX8* were cloned (Fast Pfu DNA polymerase, Novoprotein) into the *pCAMBIA1381* vector upstream of the GUS reporter gene as described previously [23], primers used in all PCRs were listed in Additional file 1.

### Transient expression procedure

Recombinant binary vectors containing the different gene constructs were transformed into *Agrobacterium* EHA105. *Agrobacterium* liquid cultures were grown at 28 °C overnight in YEP medium containing 50 μg/mL kanamycin and 50 μg/mL rifampicin until the OD_600_ reached between 1.2 and 1.5. Cells were collected by centrifugation (2100 g, 15 min) and re-suspended to an OD_600_ 0.8–1.0 in transformation solution: 1/2 Murashige and Skoog (MS) medium [24] (pH 5.8; Life Technologies), 165 μM acetosyringone (AS), 3% (w/v) sucrose, and 0.01% (v/v) Tween 20. Before use, the transformation solution (without Tween 20) was degassed using a diaphragm-type dry vacuum pump (Ulvac Kiko Inc., Kanagawa, Japan) under 0.7 kg/cm^2^ pressure for 3 min.

**Fig. 1:**
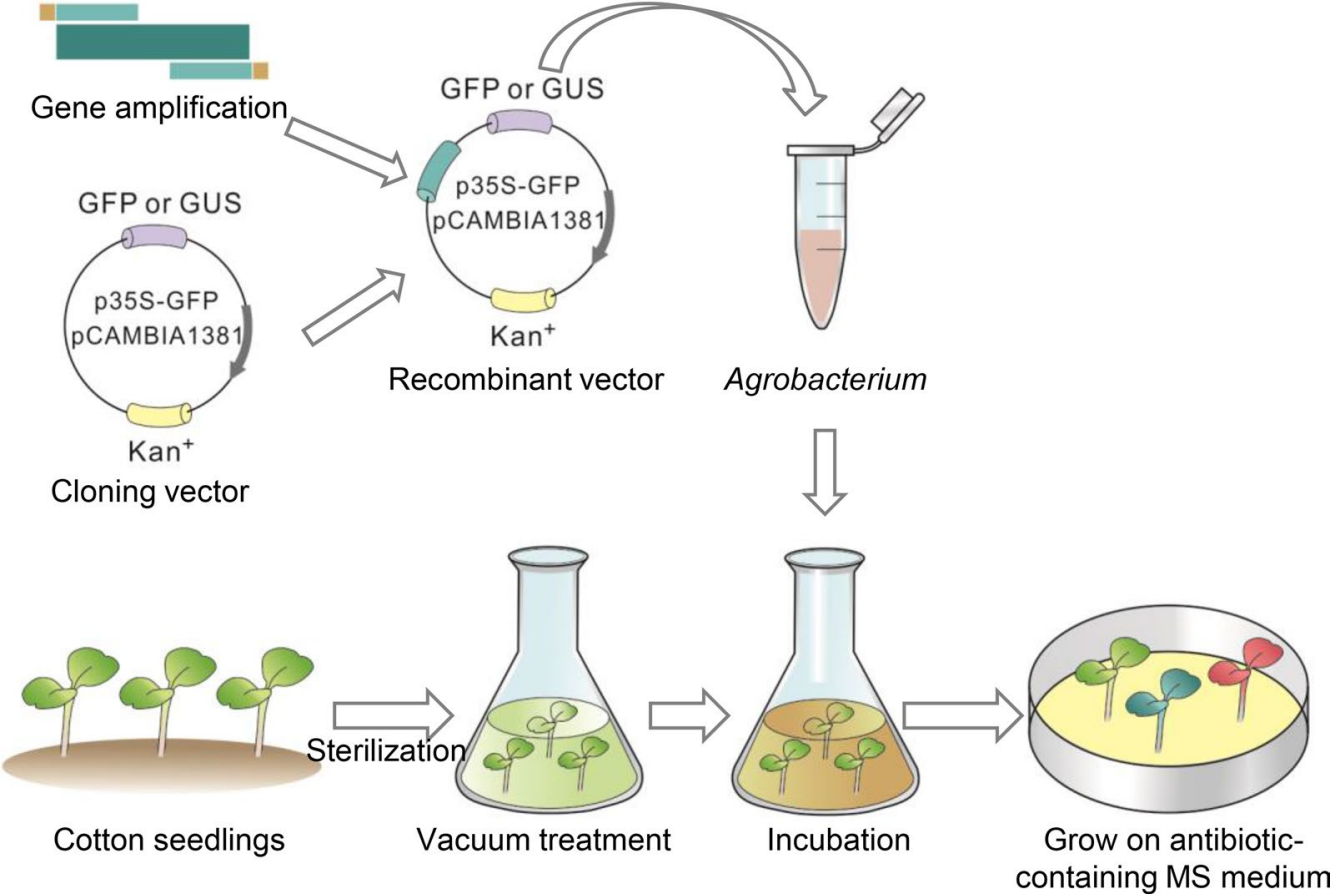
Procedure for *Agrobacterium*-mediated transient transformation of *G. hirsutum*

Seven-day-old seedlings of *G. hirsutum* were washed in double-distilled water (ddH_2_O), and sprayed with 70% ethanol. The seedlings were then immersed in 0.1% (w/v) HgCl for 2 min (If the sterilizing time is more than 2 min, the seedling would be harmed or stressed) and washed with sterile ddH_2_O five times, for 2 min each time. The aseptic seedlings were placed in sterile wide-necked bottles filled with vacuum treated transformation solution (without Tween 20) for 2 h, and seedlings were put in a sterile petri dish for vacuum infiltration using a vacuum pump under 0.7 kg/cm^2^ pressure for 2 min, then the seedlings were placed in the same sterile wide-necked bottles filled with vacuum treated transformation solution (with Tween 20) containing *Agrobacterium* and shaken at 25 °C for 5 h (120 rpm). After the co-cultivation, the seedlings were washed twice with sterile ddH_2_O, for 2 min each time and transferred onto 1/2 MS medium, pH 5.8, 0.8% (w/v) agarose that contained kanamycin 50 μg/mL, and rifampicin 50 μg/mL and kept under long-day conditions (16 h/8 h light/dark photoperiod) with light intensity of 80–90 μmol m^−2^ s^−1^ and 26/20 °C day/night temperatures. After 5 days, plants were used to analyze gene transcript levels and protein localization. Figure 1 summarizes the procedure to generate transient transgenic cotton lines, and photos of the experimental process are shown in Additional file 2.

### Gene expression analyses

Total RNA was extracted from tissues of *G*. *hirsutum* using an RNAprep Pure Plant Kit (Polysaccharides & Polyphenolics-rich, Tiangen, Beijing, China). The cDNA was synthesized using M-MLV Reverse Transcriptase (Promega, Madison, WI, USA) and diluted 30-fold with deionized water before use as template for qRT-PCR. The PCR reaction was performed on a 7500 Fast Real-Time PCR System (Applied Biosystems, Palo Alto, CA, USA) using GoTaq^®^ qPCR Master Mix kit (Promega). Transcript levels of *GhGPX1* and *GhGPX8* were normalized against an internal reference genes, *GhUBQ1* (*ubiquitin 1*, accession number: EU304080) and *GhUBQ7* (*ubiquitin 7*, accession number: DQ116441). The delta-delta Ct method was used for relative quantification data analysis in qRT-PCR experiments. At least three different sections of a single cotyledon from one seedling were processed separately as three technical replicates, and three different seedlings were used to make three biological replicates. All experiments for gene expression analysis were repeated at least for three independent biological and technical replicates were analyzed by qRT-PCR. All primers used in this study are listed in Additional file 1.

### Histochemical detection of GUS activity

For GUS assays, excised tissues from cotton plants transiently expressing the *GhGPX1* and *GhGPX8* promoter-GUS construct were stained as previously described [25]. The GUS activity of the transformed cotton seedlings was determined according to Jefferson et al. [26]. For GUS activity assays, at least three different sections of a single cotyledon from one seedling were processed separately as three technical replicates, and three different seedlings were used to make three biological replicates.

### Subcellular localization analyses

Transient expression of the recombinant vectors *p35S*-*GhGPXs*-*GFP* and control vector *p35S*-*GFP* was performed in *G*. *hirsutum* seedlings. GFP fluorescence was observed under a laser scanning confocal microscope (LSCM 710, Zeiss) with excitation at 488 nm and emission at 500–560 nm. Chloroplast autofluorescence was detected at 650–750 nm.

## Results

### Development of the transient expression method in *G. hirsutum* seedlings

**Fig. 2:**
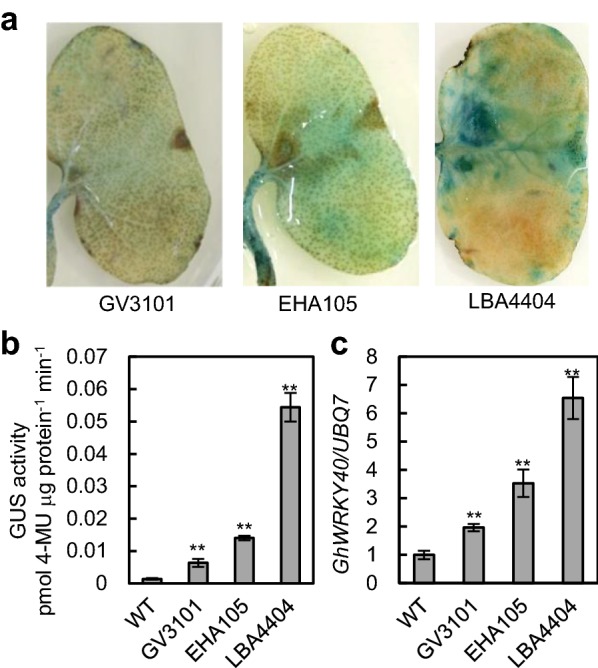
Expression patterns of *GhGPX1* promoter-GUS constructs and quantification of wound-inducible *GhWRKY40* expression levels in *G. hirsutum* plants inoculated with different *Agrobacterium* strains. GUS staining (**a**) and GUS activity in cotyledons (**b**) of plants transiently expressing *GhGPX1* promoter-GUS constructs using *Agrobacterium* EHA105, GV3101 and LBA4404 strains; at least three independent seedlings were used for GUS staining; GUS activity values are the means of approximately 5 seedlings. **c** qRT-PCR analysis of wound-inducible *GhWRKY40* gene transcript levels in cotyledons of WT and transiently expressing seedlings. The reference gene *GhUBQ7* was used for normalization purposes in the qRT-PCR assays. For each sample of GUS activity assay, at least three different sections of a single cotyledon from one seedling were processed separately as three technical replicates, and three different seedlings were used to make three biological replicates. Asterisks indicate significant differences from WT at **p < 0.01 (two tailed Students *t* test)

Various factors can influence *Agrobacterium*-mediated transformation efficiency, including bacterial strain, *Agrobacterium* cell density, co-cultivation time, protein expression kinetics, chemicals and surfactants [8, 27]. To test the efficiency of the method we selected two previously characterized genes, *GhGPX1* and *GhGPX8* [21]. The promoter regions (~ 2 kb) of each gene were cloned upstream of GUS in the *pCAMBIA1381* vector to make the recombinant plasmids, *GhGPX1pro*-*GUS* and *GhGPX8pro*-*GUS*. We first evaluated the transient expression efficiency of different *Agrobacterium* strains. *GhGPX1pro*-*GUS* was introduced into three commonly used *Agrobacterium* strains GV3101, EHA105 and LBA4404. GUS staining of inoculated cotton cotyledons showed the strongest *GhGPX1pro*-*GUS* expression in LBA4404-inoculated plants, and the lowest in GV3101-inoculated plants (Fig. 2a). Consistent with this result, cotyledons of seedlings transiently transformed with LBA4404 showed the highest GUS activity, ~ 4-fold higher than EHA105, and 10-fold higher than GV3101-inoculated plants (Fig. 2b). It is well known that *Agrobacterium* infection can cause wound-associated responses [28]. To study the extent of the wound-response caused by each of the three *Agrobacterium* strains tested, we measured the expression levels of the wound-inducible gene *GhWRKY40* [29]. As shown in Fig. 2c, LBA4404 infection caused a ~ 7-fold increase in the *GhWRKY40* expression levels compared to WT, while EHA105- and GV3101-mediated transformation produced lower levels of *GhWRKY40* expression with 4-fold and 2-fold increases over WT respectively. Therefore, we chose to use EHA105 for the development of the transient transformation system as a compromise to obtain meaningful expression levels while minimizing wound-induced gene expression.

To rule out that the observed GUS signal was not due to *GUS* expression from residual bacteria within the plant tissues, microscopic observations were performed on GUS stained tissues. Our microscopy analysis shows GUS staining confined to plant cells in stem, root and leaf samples (Additional file 3a–c). Moreover, GUS staining of a EHA105 culture containing the *GhGPX1pro*-*GUS* vector did not show any blue color (Additional file 3d).

**Fig. 3:**
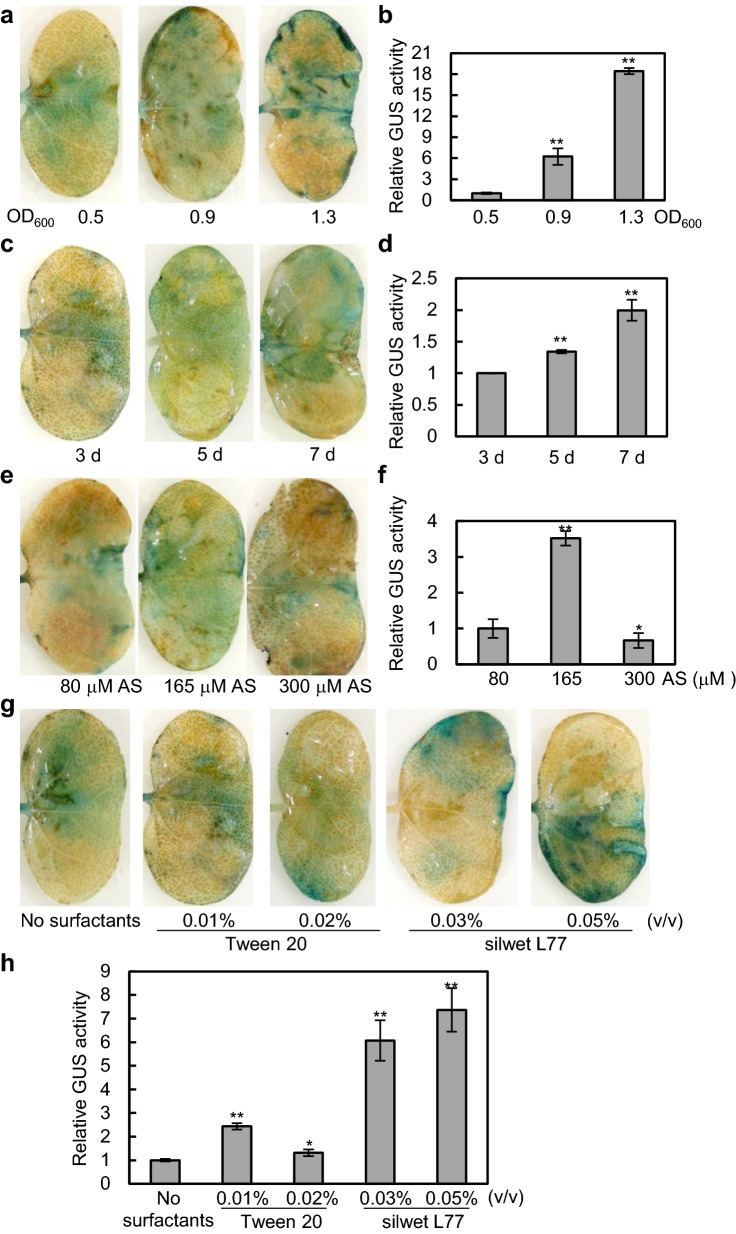
Expression patterns of *GhGPX1* promoter-GUS constructs in EHA105-inoculated *G. hirsutum* plants under different transformation condition. GUS staining (**a**, **c**, **e**, **g**) and relative GUS activity (**b**, **d**, **f**, **h**) of plants transiently expressing *GhGPX1* promoter constructs using *Agrobacterium* EHA105; **a**, **b**, effect of different *Agrobacterium* concentrations; **c**, **d**, *GhGPX1* expression levels at different times after transformation; **e**, **f**, effect of different acetosyringone (AS) concentrations; **g**, **h**, effect of different types and concentrations of surfactants. At least three independent seedlings were used for GUS staining. GUS activity values are the means of approximately 5 seedlings. The GUS activity of plants transformed with an *Agrobacterium* density of OD_600_ = 0.5 (**a**), infected for 3 days (**b**), using an AS concentration of 80 μM (**c**) and no surfactants (**d**) were given an arbitrary value of 1. For each sample of GUS activity assay, at least three different sections of a single cotyledon from one seedling were processed separately as three technical replicates, and three different seedlings were used to make three biological replicates. Asterisks indicate significant differences from WT at *p < 0.05 and **p < 0.01 (two tailed Students *t*-test)

To determine the optimal parameters for transient expression, the effect of *Agrobacterium* density was studied using EHA105 carrying the *GhGPX1pro*-*GUS* plasmid in cotton seedlings. Three *Agrobacterium* densities were tested, OD_600_ = 0.5, 0.9 and 1.3, with GUS signal being weak at OD_600_ = 0.5, and progressively increasing with higher bacterial densities (Fig. 3a, b). However, the expression levels of *GhWRKY40* increased nearly 2-, 4-, and 6-fold with *Agrobacterium* densities of OD_600_ = 0.5, 0.9 and 1.3, respectively (Additional file 4a). Although strong GUS activity was detected in seedlings inoculated with an OD_600_ = 1.3, these seedlings were often susceptible to bacterial overgrowth and showed high wound-induced gene expression.

To determine the kinetics of protein production in our method, we performed GUS staining and measured GUS enzymatic activity at different times after inoculation of cotton seedlings using *GhGPX1pro*-*GUS*. GUS staining was visible 3 days after transformation and steadily increased until day 7 (Fig. 3c) and GUS activity assays confirmed the staining results with day 7 showing double the amount of activity than at day 3 (Fig. 3d). The expression levels of *GhWRKY40* increased nearly 2-, 3-, and 7-fold at days 3, 5, and 7 after *Agrobacterium* transformation respectively (Additional file 4b). Thus, we considered 5 days after infection as the optimal time for functional studies.

We also optimized the concentrations of different transformation components such as AS and surfactants using *GhGPX1pro*-*GUS*. Three different AS concentrations, 80, 165 and 300 μM, were used in transient assays with GUS staining and activity being highest at 165 μM (Fig. 3e, f), meanwhile the *GhWRKY40* transcript levels did not show any significant differences among the three AS concentrations (Additional file 4c). The effect of different concentrations of two surfactants, Tween 20 and silwet L77, on GUS expression was tested. Our results showed that the presence of either 0.03 or 0.05% (v/v) silwet L77 in the transformation buffer produced a dramatic increase in GUS expression (Fig. 3g, h), but it also resulted in a strong increase in *GhWRKY40* expression levels (Additional file 4d). The addition of 0.01% (v/v) Tween 20 to the transformation buffer increased GUS activity significantly (Fig. 3g, h) and had no effect in the *GhWRKY40* expression levels compared with the use of no surfactants (Additional file 4d). Given these results, we chose the use of 165 μM AS and Tween 20 as the optimal parameters for our transient transformation system.

Vacuum pre-infiltration of seedlings with transformation solution before *Agrobacterium* inoculation is important to maximize *Agrobacterium* growth. We tested whether inclusion of *Agrobacterium* in the pre-infiltration solution improved efficiency but seedlings pre-infiltrated with buffer containing *Agrobacterium* died within 2 days of inoculation (Additional file 5). We also investigated whether the use of the antibiotic cefotaxime in the media to control *Agrobacterium* overgrowth could improve efficiency. We tested three different experimental conditions using (a) cefotaxime-containing media; (b) growth in rifampicin and kanamycin containing media for 5 days; and (c) initial growth in rifampicin and kanamycin containing media for 3 days followed by transfer to cefotaxime-containing media for 2 days (Additional file 6). The strongest GUS staining intensity was observed when seedlings were continuously grown on rifampicin and kanamycin containing media, while no staining was observed when seedlings were grown on cefotaxime-containing media (Additional file 6).

To determine the universality of the method, we performed transient assays in seedlings of three different cotton varieties, TM-1, CCRI36 and Coker201 using *Agrobacterium*, strain EHA105 containing the *GhGPX1pro*-*GUS* vector. GUS staining was visible in all three cultivars (Additional file 7) with the strongest staining observed on the TM1 variety which was chosen for all subsequent experiments.

### Generation of *G. hirsutum* seedlings with strong transgene expression

*Agrobacterium tumefaciens,* EHA105 strain, carrying the *GhGPX1pro*-*GUS* or *GhGPX8pro*-*GUS* plasmids were used for the transient transformation of *G. hirsutum* seedlings by the procedure developed in this study and plants were grown on antibiotic-containing (kanamycin and rifampicin) MS medium. As shown in Additional file 8, the overall transformation efficiency was 57.6% (number of seedlings harboring *GhGPX1*/*GhGPX8pro*-*GUS* after 8 days/total number of seedlings infected with *Agrobacterium*).

**Fig. 4:**
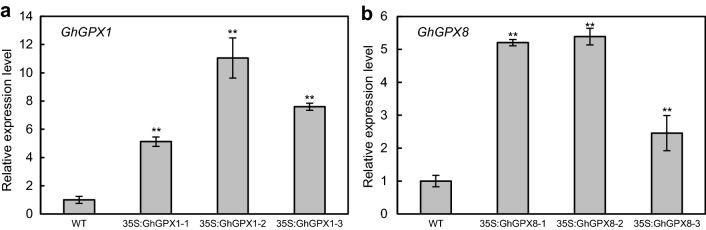
Quantification of transgene expression levels in un-inoculated controls and EHA105-inoculated *G. hirsutum* plants. *GhGPX1* (**a**) and *GhGPX8* (**b**) transcript levels in cotyledons of control and transiently expressing seedlings was determined by qRT-PCR. Three independent sets of seedlings (35S:GhGPX1-1/2/3 and 35S:GhGPX8-1/2/3) were inoculated and a minimum of six different cotyledon sections from each seedling were used for the qRT-PCR assay. Endogenous WT level for each gene was given the arbitrary value of 1. Asterisks indicate significant differences from WT at **p < 0.01 (two tailed Students *t*-test)

In order to study the expression levels achieved by our transient transformation method, the coding regions of *GhGPX1* and *GhGPX8* were fused in frame with *GFP* under the control of the CaMV 35S promoter generating the *p35S*-*GhGPX1*-*GFP* and *p35S*-*GhGPX8*-*GFP* plasmids. Seedlings subjected to transient transformation were analyzed by qRT-PCR using the endogenous reference gene *GhUBQ7* as control (accession number: DQ116441), showing high expression levels of the transgenes (Fig. 4a, b). The *GhGPX1* transcript levels were 5–11 times higher than those observed for untransformed controls (Fig. 4a) while *GhGPX8* showed 3–5 times higher levels than controls (Fig. 4b). To confirm our results a second endogenous reference gene, *GhUBQ1* (accession number: EU304080), was used in the qRT-PCR assays with similar results (Additional file 9). These results confirmed the success of our method in generating cotton plants transiently overexpressing target genes.

### Characterization of the *GhGPX1* and *GhGPX8* promoters

**Fig. 5:**
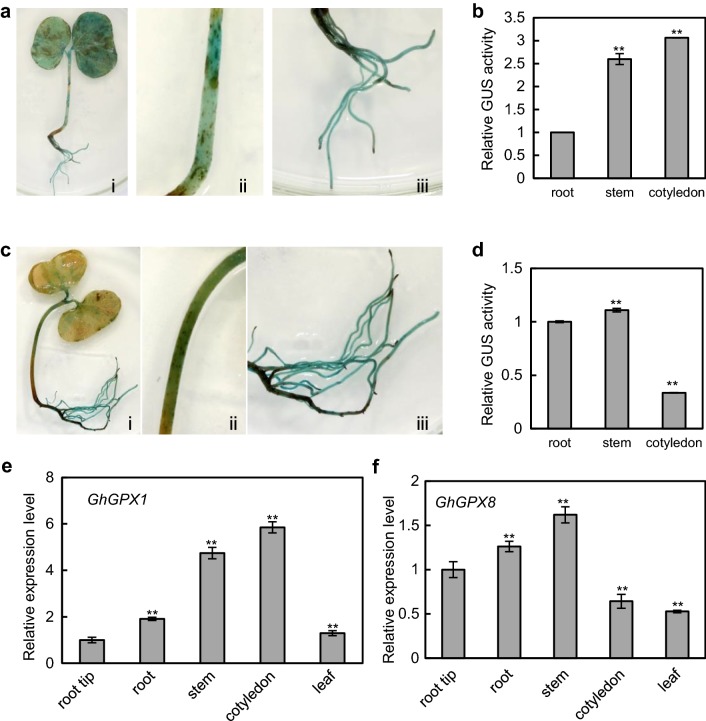
Expression patterns of *GhGPX1* and *GhGPX8* promoter-GUS constructs in *G. hirsutum* plants. GUS staining of plants transiently expressing *GhGPX1* and *GhGPX8* promoter constructs (**a**) and (**c**); expression patterns in whole seedlings (i), stems (ii), and roots (iii) were visualized by histochemical GUS staining. Relative GUS activity in root, stem and cotyledon of plants transiently expressing *GhGPX1* (**b**) or *GhGPX8* (**d**) promoter constructs. **e**, **f** Quantification of endogenous *GhGPX1* and *GhGPX8* transcript levels by qRT-PCR in WT non-inoculated plants. For each experiment, tissue from three different 12-d-old *G. hirsutum* seedlings was collected. For all experiments on gene expression analysis, at least three different sections of each tissue from one seedling were processed separately as three technical replicates, and three different seedlings were used to make three biological replicates were performed. Asterisks indicate significant differences from root tip at **p < 0.01 (two tailed Students *t*-test)

Seedlings transiently transformed with the *GhGPX1pro*-*GUS* and *GhGPX8pro*-*GUS* constructs were studied to characterize the expression patterns driven by the promoters of the two genes. Histochemical staining revealed that *GhGPX1pro*-*GUS* was expressed throughout the plant, including cotyledons (Fig. 5a. i), stems (Fig. 5a. ii) and roots (Fig. 5a. iii), with the strongest staining detected in the cotyledons. GUS activity assays confirmed the histochemical observations with cotyledon and stem GUS activity values being 3- and 2.5-fold higher than root respectively (Fig. 5b). Whole seedlings transformed with *GhGPX8pro*-*GUS* showed strong staining in stems (Fig. 5c ii) and roots (Fig. 5c iii), while staining in cotyledons (Fig. 5c i) was weaker. Again, GUS activity assays confirmed the histochemical observations (Fig. 5d). These results are consistent with the *GhGPX1* and *GhGPX8* endogenous transcript levels quantified by qRT-PCR in untransformed *G. hirsutum* plants (Fig. 5e, f), indicating that the *Agrobacterium*-mediated transient transformation method was suitable for promoter characterization in cotton.

### GhGPX1 and GhGPX8 are localized in the chloroplast and cytoplasm, respectively

**Fig. 6:**
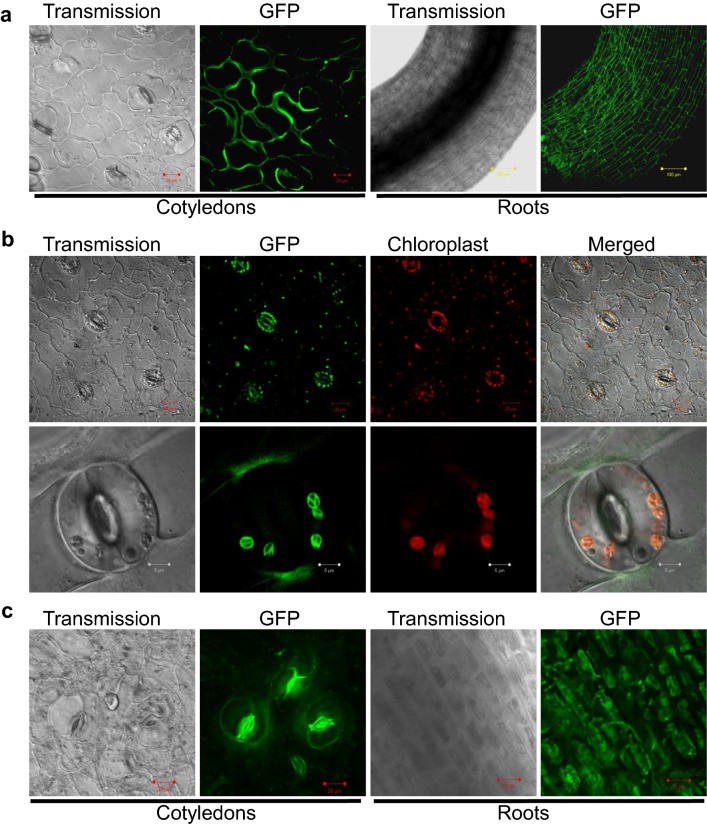
Subcellular localization of GFP-tagged GhGPX1 and GhGPX8 in *G. hirsutum*. **a** Transient expression of control vector *pro35S*-*GFP*. **b** Transient expression of the *Pro35S*-*GhGPX1*-*GFP* construct resulted in strong GFP fluorescence in the chloroplasts of epidermal and guard cells detected by confocal microscopy. Pictures in the bottom line show a close up of a guard cell. **c** Confocal microscopy of plants transiently expressing the *Pro35S*-*GhGPX8*-*GFP* construct shows fluorescence in the cytosol of epidermal and root cells. White bars, 5 μm; red bars, 10 μm; yellow bars, 100 μm. At least 7 different seedlings from three independent biological and technical replicates were used for each experiment with similar results

We also determined whether our method is suitable for intracellular localization studies by analyzing the GFP fluorescence patterns in the epidermis of seedlings transiently expressing either GhGPX1-GFP or GhGPX8-GFP fusion proteins. Compared with the control transformed with the empty p35S-GFP vector (Fig. 6a), transient transformation with the *p35S*-*GhGPX1*-*GFP* construct produced a strong fluorescence signal confined to chloroplasts and especially strong in guard cells (Fig. 6b), consistent with our previous finding that GhGPX1 was located in the chloroplasts of *Arabidopsis* protoplasts [21]. Seedlings expressing GhGPX8-GFP showed strong fluorescence in the cytoplasm of cotyledon and root cells (Fig. 6c), also consistent with our previous results showing that GhGPX8 was located in the cytoplasm of *Arabidopsis* protoplasts [21]. These results confirmed that the transient transformation method was suitable for subcellular localization studies in cotton.

## Discussion

We have developed a transient expression system to perform rapid and technically easy gene characterization studies in *G. hirsutum*. Plant transformation is a core research tool for cotton improvement as well as gene functional studies. Although, several transformation methods have been developed and refined to increase transformation efficiency and stably express transgenes in cotton, they are technically complex and time-consuming [30].

*Agrobacterium*-mediated transient transformation is a complex process with many environmental and biological factors affecting its efficiency such as the composition of the culture medium, the binary plasmid vector, the host plant and the *Agrobacterium* strain [9, 29–33]. We established the optimal parameters to obtain high efficiency as well as high expression levels of the target gene in *G. hirsutum*. The choice of optimal conditions for the assay needs to take into account the purpose of the experiment. A number of factors have a dramatic effect on the efficiency of the transformation system as well as the levels of expression achieved but can also result in secondary and unwanted effects. For purposes, such as promoter studies and overexpression of transgenes, the *Agrobacterium* strain EHA105 was selected because it produced good GUS activity levels, although not the highest, and a moderate wound response (Fig. 2). Similar consideration was given to the *Agrobacterium* concentration, with OD_600_ 0.8–1.0 resulting in good efficiency, while higher concentrations led to seedling death and low concentrations resulted in few or no plants showing transient expression; the optimal co-cultivation period was 6 h, with shorter or longer incubation time negatively affecting efficiency and/or leading to seedling browning or death (Additional file 10). Previous studies have shown that stable expression requires T-DNA integration into the host genome, and that the use of selection results in an increase in transgene expression levels 10–14 days post infection of plant tissues [34]. Indirect evidence indicates that transient expression predominantly occurs from T-DNA copies that are not integrated into the host genome [35, 36]. Transient expression usually peaks 2–4 days post infection before declining in the number of expressing cells as well as the expression level per transformed cell but the kinetics ultimately depends on the host plant genotype [35]. In this study, we observed that gene transcript levels varied over time and selected materials at 5 days post infection for the analyses of gene expression studies (Fig. 3c, d; Additional file 4b). For subcellular localization characterization, *Agrobacterium* GV3101 gave similar results to EHA105 (Additional file 11). Moreover, for localization studies, where secondary wounding effects do not influence the localization of most cellular proteins, different optimal parameters should be chosen such as the use of the LBA4404 strain, higher *Agrobacterium* density and silwet L77 as surfactant. We observed that wounding enhanced the transformation efficiency as well as the transcript levels of the transgenes (Fig. 3; Additional file 4). However, wounding significantly affects the steady state expression levels of many genes and therefore the wound-related results have not been included in this manuscript.

Our *Agrobacterium*-mediated transient transformation method for upland cotton is fast and easy to scale up to produce relatively large amounts of recombinant protein. Aside from the applications shown in this work, the method could also be used for protein–protein interaction studies and other research requiring the expression of genes in cotton. In future work, we will focus on further enhancing the transformation efficiency and performing gene functional studies in cotton.

## Conclusion

We have developed a fast and easy *Agrobacterium*-mediated transient transformation method for upland cotton. Our method can be used for gene functional studies such as promoter characterization and protein subcellular localization in cotton, obviating the need to perform such studies in a heterologous system such as *Arabidopsis*.

## Additional files

**Additional file 1.** Primers used in this study.

**Additional file 2.** Pictorial representation of the *Agrobacterium*-mediated transient transformation of *G. hirsutum*.

**Additional file 3.** Transient GUS expression in cotton plants by *GhGPX1pro*-*GUS*-contained EHA105.

**Additional file 4.** Relative *GhWRKY40* expression levels in EHA105 *Agrobacterium*-inoculated *G. hirsutum* plants under different transformation conditions.

**Additional file 5.** Transient transformation of cotton seedlings with *GhGPX1pro*-*GUS* under vacuum infiltration with or without *Agrobacterium* in the transformation solution.

**Additional file 6.** GUS staining of transiently transformed cotton seedlings.

**Additional file 7.** GUS staining of *G. hirsutum* cultivars CCRI36, Coker201 and TM-1 after transient transformation with *A. tumefaciens* EHA105 containing the *GhGPX1pro*-*GUS* vector.

**Additional file 8.** Transformation efficiency.

**Additional file 9.** Quantification of relative expression levels for *GhGPX1* and *GhWRKY40* in *Agrobacterium*-inoculated *G. hirsutum* plants using two different endogenous reference genes.

**Additional file 10.** GUS staining of plants transiently expressing *GhGPX1* promoter constructs using *Agrobacterium* EHA105-mediated transient transformation at different times after inoculation.

**Additional file 11.** Subcellular localization of GFP-tagged GhGPX1 in *G. hirsutum* plant leaves using *Agrobacterium* GV3101-mediated transient transformation.

## Abbreviations

*G. hirsutum*: *gossypium hirsutum*
CGP: cotton genome project
qRT-PCR: quantitative real-time reverse transcriptase polymerase chain reaction
T-DNA: transferred DNA
PCR: polymerase chain reaction
GFP: green fluorescent protein
GUS: β-glucuronidase
GPX: glutathione peroxidase
AS: acetosyringone
