# Supplementary material for: A transient transformation system for gene characterization in upland cotton (Gossypium hirsutum)
Source: Plant Methods. 2018 Jun 22;14:50. doi: 10.1186/s13007-018-0319-2 (PMC6013946; doi:10.1186/s13007-018-0319-2)
Supplement: Supplementary file 1 — Additional file 1. Primers used in this study. [file 13007_2018_319_MOESM1_ESM.docx]

**Additional file 1. Primers used in this study.**

| Genes |  | Sequence |
| --- | --- | --- |
| **qRT-PCR** |  |  |
| *UBQ1* | F | 5' CCAGAAGGAATCCACTTTGC 3' |
|  | R | 5' CCAGCTCACATCAGCATACG 3' |
| *UBQ7* | F | 5' GAAGGCATTCCACCTGACCAAC 3' |
|  | R | 5' CTTGACCTTCTTCTTCTTGTGCTTG 3' |
| *GhGPX1* | F | 5' CTGCTTCTGGGTTTGTCTTTAAC 3' |
|  | R | 5 'GGGTATCCTTCCCATCAATATCC 3' |
| *GhGPX8* | F | 5' AGACAGAGCCCGTCTACAA 3' |
|  | R | 5' TGGCCATCCTTGTCAACTAAA 3' |
| *GhWRKY40* | F | 5' AACACAACCATCTTCCTCCTTCTC 3' |
|  | R | 5' TTTGCCGAAGTCTGGAGTCA 3' |
| ***pCAMBIA1381::GhGPX*** |  |  |
| *GhGPX1* | F | 5' CCCGGATCCGTAGTGGAAGGTAAAAGTCCA 3' (*Bam*H I) |
|  | R | 5' GGGCTGCAGAAGACGCACAATAAAACCTT 3' (*Pst* I) |
| *GhGPX8* | F | 5' CCCGGATCCGTTATGGAACGTCCTATAACT 3' (*Bam*H I) |
|  | R | 5' CCCAAGCTTTAACAGCGGAATCAGGCGAAA 3' (*Hin*d III) |
| ***p35S::GhGPX-GFP*** |  |  |
| *GhGPX1* | F | 5' CCCAAGCTTATGGCTTCCATGTCTTTCTC 3' (*Hin*d III) |
|  | R | 5' CCCGTCGACTGTCGCGAGGAGCTTCTGAA 3' (*Sal* I) |
| *GhGPX8* | F | 5' CCCAAGCTTATGGCTTCTCAATCTTCTA 3' (*Hin*d III) |
|  | R | 5' CCCGTCGACAGCCAGCAGTTTCTTAA 3' ( *Sal* I ) |
