# Supplementary material for: A transient transformation system for gene characterization in upland cotton (Gossypium hirsutum)
Source: Plant Methods. 2018 Jun 22;14:50. doi: 10.1186/s13007-018-0319-2 (PMC6013946; doi:10.1186/s13007-018-0319-2)
Supplement: Supplementary file 8 — Additional file 8. Transformation efficiency. [file 13007_2018_319_MOESM8_ESM.doc]

**Additional file 8**. Transformation efficiency.

Overall transformation efficiency 57.6%

| **Total no. of infected seedlings** | **No. of transformed**  **seedlings** | **No. of weakly transformed seedlings** | | **Mortality** | **Transformation efficiency** |
| --- | --- | --- | --- | --- | --- |
| 10 | 4 | 2 | 2 | | 40.0% |
| 10 | 5 | 1 | 3 | | 50.0% |
| 9 | 5 | 0 | 1 | | 55.6% |
| 10 | 6 | 2 | 1 | | 60.0% |
| 10 | 4 | 3 | 0 | | 40.0% |
| 10 | 7 | 3 | 0 | | 70.0% |
| 10 | 6 | 3 | 1 | | 60.0% |
| 10 | 5 | 5 | 0 | | 50.0% |
| 10 | 8 | 0 | 2 | | 80.0% |
| 10 | 7 | 3 | 0 | | 70.0% |

Total no. of infected seedlings = number of cotton plants used for transient transformation assay.

No. of transformed seedlings = seedlings expressing *GUS* in all tissues.

No. of weakly transformed seedlings = seedlings expressing *GUS* in some tissues.

Mortality = number of cotton plants that did not survive on medium containing antibiotics.

Transformation efficiency = ratio of transformed plants to total plants.

A total of 99 seedlings were used for transformation of GUS, each plant only transformed with one construct, *GhGPX1-GUS* or *GhGPX8pro-GUS*.
